# Simultaneous Surface-Enhanced Raman Scattering with a Kerr Gate for Fluorescence Suppression

**DOI:** 10.1021/acs.jpclett.3c02926

**Published:** 2024-01-10

**Authors:** Gema Cabello, Igor V. Sazanovich, Ioannis Siachos, Matthew Bilton, Beata L. Mehdi, Alex R. Neale, Laurence J. Hardwick

**Affiliations:** † Stephenson Institute for Renewable Energy, Department of Chemistry, University of Liverpool, Peach Street, Liverpool L69 7ZF, U.K.; ‡ The Faraday Institution, Quad One, Harwell Science and Innovation Campus, Didcot OX11 0RA, U.K.; § Central Laser Facility, Research Complex at Harwell, STFC Rutherford Appleton Laboratory, Harwell Campus, Didcot OX11 OQX, U.K.; ∥ Department of Mechanical Materials and Aerospace Engineering, University of Liverpool, Brownlow Hill, Liverpool L69 3GH, U.K.; ⊥ SEM Shared Research Facility, University of Liverpool, Brownlow Hill, Liverpool L69 3GH, U.K.

## Abstract

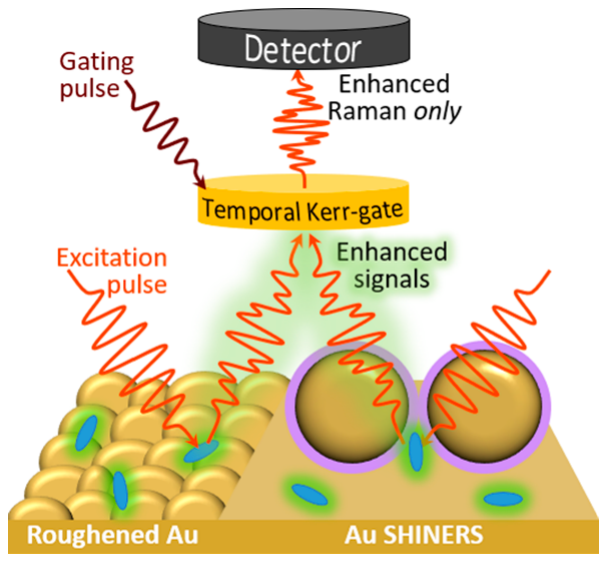

The combination of surface-enhanced and Kerr-gated Raman spectroscopy for the enhancement of the Raman signal and suppression of fluorescence is reported. Surface-enhanced Raman scattering (SERS)-active gold substrates were demonstrated for the expansion of the surface generality of optical Kerr-gated Raman spectroscopy, broadening its applicability to the study of analytes that show a weak Raman signal in highly fluorescent media under (pre)resonant conditions. This approach is highlighted by the well-defined spectra of rhodamine 6G, Nile red, and Nile blue. The Raman spectra of fluorescent dyes were obtained only when SERS-active substrates were used in combination with the Kerr gate. To achieve enhancement of the weaker Raman scattering, Au films with different roughnesses or Au-core-shell-isolated nanoparticles (SHINs) were used. The use of SHINs enabled measurement of fluorescent dyes on non-SERS-active, optically flat Au, Cu, and Al substrates.

The development of metal configurations that provide enhancement of optical fields near their surfaces, along with the control of light–matter interactions, evolved from the observation of enhanced Raman signals of pyridine molecules adsorbed onto a roughened silver electrode by Fleischmann.^1^ What became known as surface-enhanced Raman spectroscopy (SERS)^2,3^ was initially restricted to the use of some free-electron-like metals that can generate optical conductive resonance in the visible spectral range (Group 1B and alkali metals).^4−6^ More recently, this field has grown further into the vast field of core-shell nanoparticle-enhanced Raman spectroscopy that includes shell-isolated nanoparticles for enhanced Raman spectroscopy (SHINERS).^7−9^ Hybrid nanoplasmonic systems, i.e., the combination of plasmonic nanomaterials with catalytic metals, semiconductors, metal–organic frameworks, and many others, have extended the application of Raman spectroscopy to nearly every imaginable substrate/nanomaterial-target analyte configuration ever since.^10−12^

To further broaden the application of Raman spectroscopy, the challenge of fluorescence interference must be overcome. Fluorescence may arise from the analyte itself, as well as matrix components, sample/solvent contaminants, or the components in the optical setup or be generated from the chemical or electrochemical process under investigation. In a majority of systems, the broad fluorescence background cannot be effectively subtracted from the Raman signal once it becomes significantly large.^13^ Raman scattering and fluorescence emission are two contending phenomena but differ in time scales and in the nature of the intermediate states. Raman scattering is a virtually instantaneous process, in which the interaction of the photon with the molecule and photon scattering occur almost simultaneously (≤10^–12^ s) through virtual states. In contrast, luminescence emission follows an absorption/relaxation process from the ground and excited electronic states, which usually requires >10^–9^ s. Because luminescence emission is statistically relatively slower than Raman scattering, the fluorescence background can be reduced in the collected Raman spectra by effectively time-gating the signal.^14^ Fluorescence rejection techniques have been steadily developed over the past 50 years, including the development of picosecond optical Kerr-gated detection^15−18^ and other time-gated variations.^19,20^ Kerr-gated Raman spectroscopy allows for the rejection of the pure fluorescence background from the Raman spectrum observed with a detection time gate coincident with the Raman signal (when the fluorescence emission is from an electronic state whose lifetime is long enough relative to the laser pulse duration and the detection time).^21^ Kerr-gated Raman spectroscopy has been successfully applied for a variety of research fields, such as catalysis, the biomedical field, sensing, and energy conversion and storage.^22−28^ However, its performance is still limited to analytes with a large Raman cross section, which, again, restricts the use of Raman spectroscopy as a routine analytical method.

Herein, the combination of surface enhancement effects and Kerr-gated Raman spectroscopy for the enhancement of the Raman signal and suppression of fluorescence is reported utilizing SERS/SHINERS. Shell-isolated nanoparticles (SHINs) have been extensively used since their development in 2010 as electromagnetic resonators on non-SERS substrates to enhance the electric field of the incident electromagnetic radiation.^9,29^ The SHINs used here were synthesized with an average Au core diameter of 50 nm, resulting in a plasmonic resonance maximum at ∼540 nm with no noticeable effects on the optical properties arising from the outer shell (Figure S1a). The role of the shell, typically SiO_2_ as used here, is to protect the metallic core (Au herein) from exposure to the media and/or aggregation and to inhibit surface chemical or electrical interactions with the analyte or other parts of the system under investigation.^30,31^ The SHINs are utilized herein in conjunction with different surface substrates (with varying inherent signal enhancement properties) to demonstrate the effective coupling of signal enhancement effects and fluorescence rejection. Thus, simultaneous SHINERS/SERS and Kerr-gated Raman spectroscopy in multiple configurations enable the measurements of challenging materials (and concentrations) that could have wide applicability for many fields in which a large emission background obscures surface relevant peaks.

The 633 nm excitation line used throughout this work has been demonstrated as an appropriate wavelength for achieving scattering enhancement interactions with ∼50 nm Au nanoparticles, without being dominated by absorption that coincides more closely with the extinction maximum.^32^ In this regard, the absorption spectra of SHINs deposited on glass (i.e., not in solution) reveal the shift in the absorbance maximum (Figure S2a, ∼603 nm) as a result of particle interactions that do not occur in liquid dispersions. Likewise, absorbance spectra of SHINs deposited on the Au film substrate (described later in this work) show similar evidence of a plasmonic resonance maximum arising from the SHINs centered around ∼600 nm (Figure S2b,c). However, even for a comparatively thin Au film (20 nm) on glass with excess SHINs deposited, necessitated by this transmission measurement configuration, the absorption spectra were dominated by the Au film, and evidence for the absorption maximum of the SHINs layer was derived only by difference spectra (Figure S2c).

Initially, the combination of SHINERS with Kerr-gated Raman spectroscopy was investigated using rhodamine 6G {Rh6G, 9-[2-(ethoxycarbonyl)phenyl]-*N*-ethyl-6-(ethylamino)-2,7-dimethyl-3*H*-xanthen-3-iminium chloride} under a 633 nm excitation line. The dye molecule, Rh6G, is regularly used as a probe molecule in Raman spectroscopy as Raman signals of chemisorbed Rh6G are obtained under preresonant excitation (Figure S3a, pink). Accordingly, the occurrence of an increased SERS enhancement factor has been suggested.^33,34^ The strong S1–S0 absorption of Rh6G (0.5 nM in ethanol) displayed a peak maximum of 530 nm and a vibronic shoulder at 490 nm (Figure S3a, pink).

The resonance Raman spectrum of solid Rh6G as a reference is presented in Figure 1a-i. The preresonant condition allowed the Raman spectrum to be revealed for the solid sample and diluted solutions (Figure 1a-ii and -iii) without the interference of fluorescent emission (emission maximum at 590 nm).^35^ High-frequency bands with large scattering cross sections were assigned to the C–H in-plane bending mode in the xanthene ring at 1184 cm^–1^, the hybrid mode (xanthene/phenyl rings and NHC_2_H_5_ group) at 1310 cm^–1^, the in-plane C–C stretching modes in the xanthene ring at 1361 and 1508 cm^–1^, the C–C stretching mode in the phenyl ring at 1572 cm^–1^, the hybrid mode (phenyl ring with COOC_2_H_5_) at 1600 cm^–1^, and the C–C stretching mode in the xanthene ring at 1648 cm^–1^.

**Figure 1:**
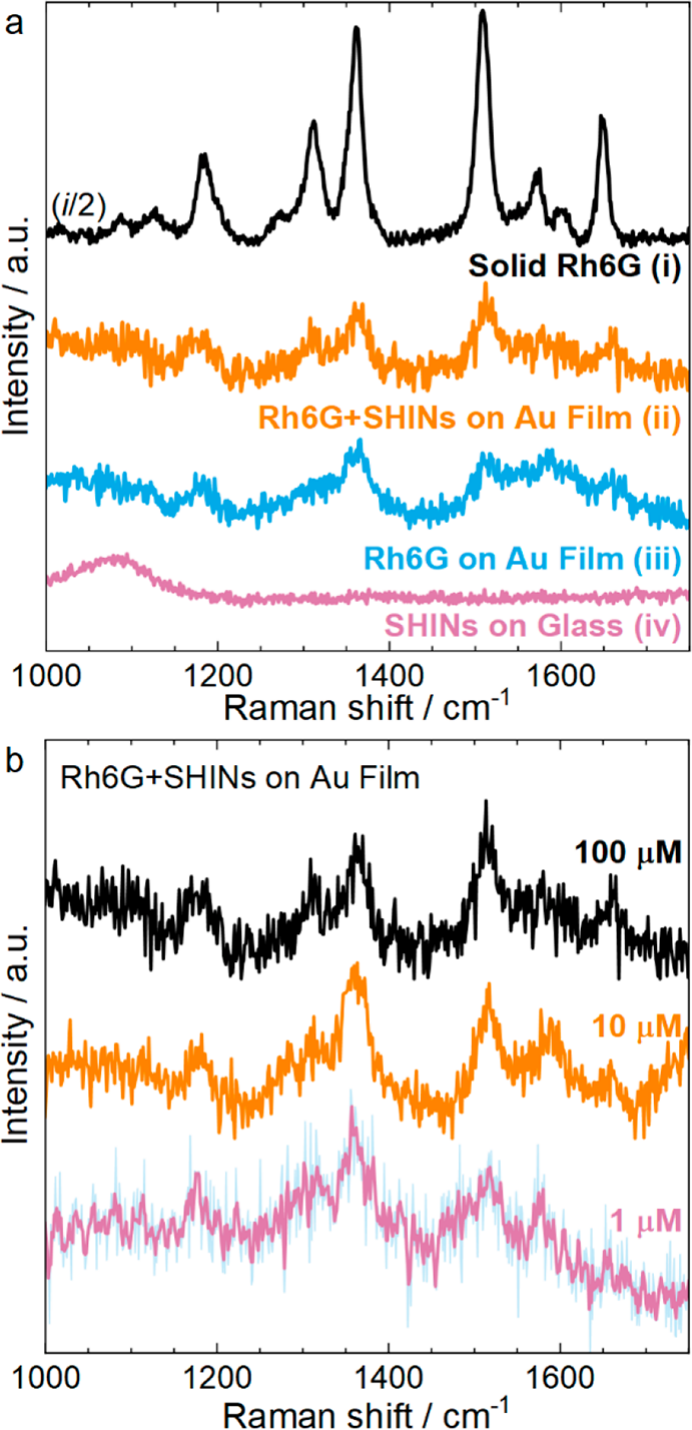
(a) (i) Resonance Raman scattering spectrum of solid Rh6G. Kerr-gated Raman spectra of chemisorbed Rh6G (100 μM solution) on an evaporated Au film (ii) with SHINs and (iii) without SHINs. (iv) Kerr-gated Raman spectrum of SHINs on a glass window (no Rh6G). (b) Kerr-gated Raman spectra of Rh6G chemisorbed on a Au film with a layer of SHINs at a range of Rh6G concentrations: 1 μM (pink), 10 μM (orange), and 100 μM (black). All spectra in panels a and b were recorded under a 633 nm excitation line, and mathematical smoothing was applied to the 1 μM spectra in panel b, where the faded blue trace underneath shows the unprocessed spectra. Spectrum i in panel a was obtained under continuous wave laser excitation, while all other spectra were recorded with picosecond pulsed laser excitation.

The Kerr-gated Raman spectra of chemisorbed Rh6G on an evaporated Au film (100 nm thick) with and without a layer of SHINs are shown as spectra a-ii and a-iii, respectively, in Figure 1. Figure 1a-iv shows the spectrum of SHINs on glass (in the absence of Rh6G), demonstrating that the SHINs do not introduce additional bands. The different substrates and the related preparative methods used throughout this work are described in greater depth in the Supporting Information. Herein, the evaporated Au film (in the absence of SHINs) comprised sufficient roughness to produce appreciable SERS enhancement^36^ (Figure 1a-iii), and the main Raman bands of Rh6G (i.e., 1184, 1310, 1361, 1508, and 1658 cm^–1^) were clearly identified in both cases. The SERS activity from the substrate alone canceled the expected enhancement of the plasmonic nanoparticles (NPs), and both spectra, that from the Au film coated with SHINs and that from the bare Au film, are comparable with regard to band shape and intensity. In the first instance, one would expect a higher intensity of bands associated with Rh6G chemisorbed on the bare Au film (Figure 1a-iii) compared to that with SHINs (Figure 1a-ii) because the relative area of the Au film is obscured by the deposited SHINs. However, the reproducibility of measured signal intensities is technically challenging from sample to sample compared to the reproducibility of wavenumber positions and relative intensities within discrete spectra. The Kerr-gated SERS spectra of chemisorbed Rh6G at different concentrations are shown in Figure 1b. Well-resolved spectra were obtained, and the Raman modes at 1184, 1310, 1361, and 1508 cm^–1^ were identified down to 1 μM Rh6G.

Nile red [NR, 9-(diethylamino)-5*H*-benzo[*a*]phenoxazin-5-one] exhibits emission self-quenching in the solid state [fluorescence resonance energy transfer (FRET)] and solvatochromism (in which the color of a solution varies when the solute is dissolved in different solvents), which is more pronounced in polar solvents. Most fluorophores, including those used in this study, show variations in the quantum yield (QY) with the concentration and the nature of the solvent, especially its polarity.^37^ Changes have been attributed to dye–dye interactions, which may further affect the electromagnetic dipole–dipole interaction between adsorbed dyes on metal surfaces.^38,39^ Raman data for NR were obtained under a continuous wave (CW) 633 nm excitation line without Kerr gating [such excitation is preresonant with the emission maximum (Figure S3, red trace)] and are shown in Figure 2a. The spectrum labeled as NR-aggregate (Figure 2a, black trace) corresponds to 10 μL of 30 nM NR in acetone dried on a Au film. Weak bands are observed in this spectrum due to emission quenching caused by dimerization during aggregate formation.^40^ Main bands were identified beyond the strong background: ring II breathing + β(C-CO-C) ring III at 590 cm^–1^, ν(C-CO-C) ring III + μ(C-H) at 855 cm^–1^, β(C-H) ring IV + ν(C-N-C) ring II + β(C-H) ring I at 1180 cm^–1^, and ν(C-C) ring IV + ν(C-N19-C) ring II + ν(C-N) ring I + ν(C-C) ring I at 1450 cm^–1^. The Raman spectrum of a solution containing 30 nM NR in acetone and an ∼100 μm optical path displayed an intense background from photoluminescence (Figure 2a, orange).

**Figure 2:**
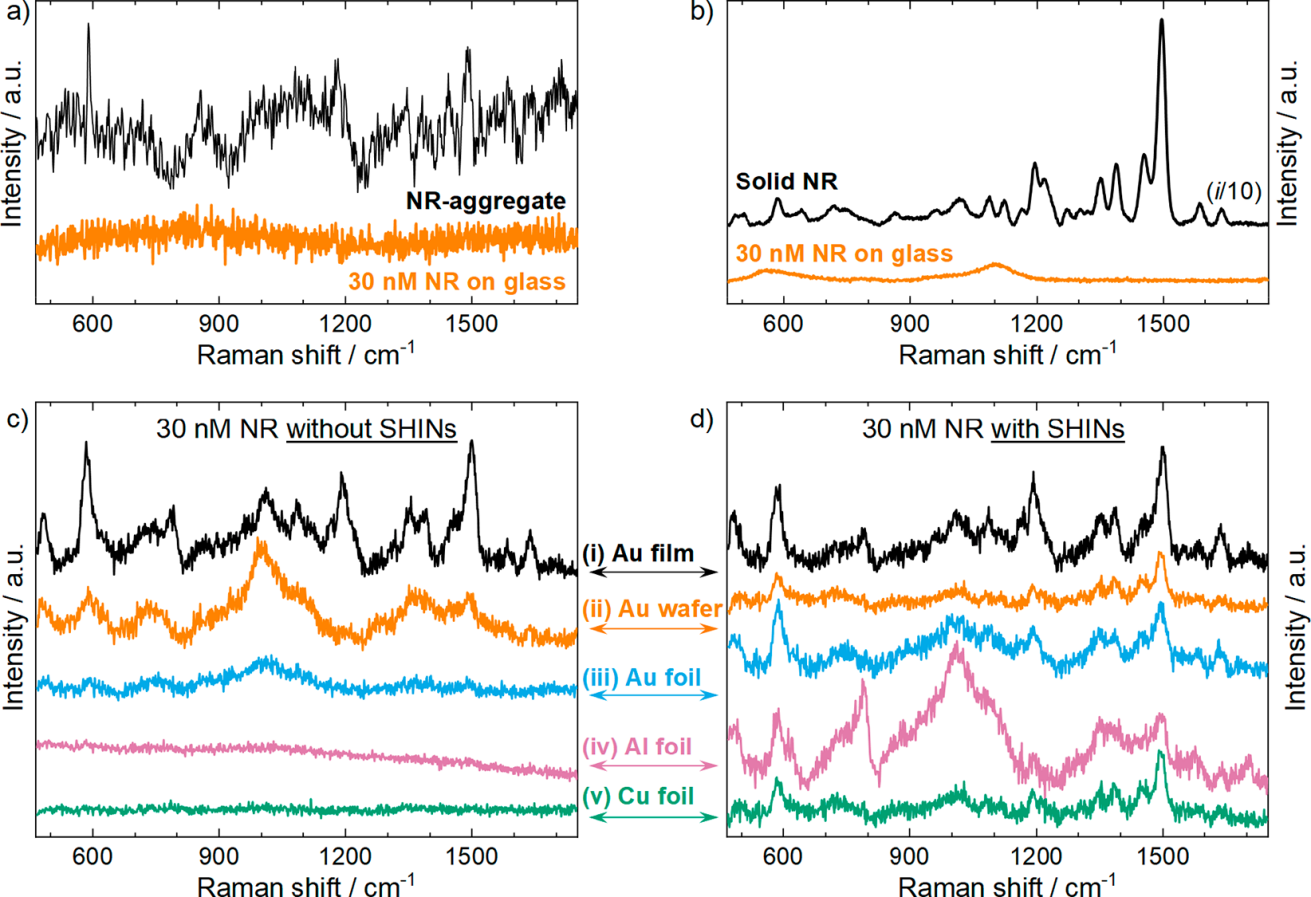
(a) Raman spectra of the Nile red aggregate (NR-aggregate, black) and a solution containing 30 nM NR in acetone on glass (orange). (b) Kerr-gated Raman scattering of solid NR (black) and a solution containing 30 nM NR in acetone on glass (orange). (c) Kerr-gated Raman spectra of a solution containing 30 nM NR in acetone on a Au film (i), a Au wafer (ii), a Au foil (iii), an Al film (iv), and a Cu film (v). (d) Same as panel c, but with all substrates containing a layer of SHINs. All spectra were collected under a 633 nm excitation line. The spectra in panel a were recorded under CW laser excitation, while all other spectra were obtained with picosecond pulsed laser excitation.

Similar experiments were completed by using Kerr-gated Raman spectroscopy (Figure 2b). Fluorescence rejection of solid NR is achieved using Kerr-gated Raman scattering (Figure 2b, black), enabling the clear identification of all of the Raman bands. The Raman band frequency–vibration correlations are described in Table S1. Using the Kerr gate, fluorescence rejection for the solution containing 30 nM NR in acetone was also achieved, but only the typical broad bands from SiO_2_ were detected originating from the underlying substrate on a glass microscope coverslip (Figure 2b, orange).

After demonstrating that Kerr-gated Raman scattering provides selective detection of Raman signals for solid NR over generally longer living fluorescence, we sought to establish the detection of fluorophores in solution. Therein, three Au substrates with different roughnesses [Au film evaporated on glass, Au on wafer (Au on a Si wafer), and Au foil] were interrogated, and the corrugation grade of the surfaces was qualitatively compared by the oxide stripping method.^29,41^ Therein, the charge in the cathodic peak on the voltammogram correlates with the surface area of the Au working electrode (Figure S4), and therefore, the roughness grade can be inferred. The roughness factor was normalized to the measured charge of the Au foil, which was considered a non-SERS-active substrate. The charge passed under the oxide reduction peak for the Au film on glass was higher than that of the other samples, resulting in a roughness factor of 5.1, whereas the Au wafer exhibited a roughness only slightly greater than that of the foil (1.7). The significantly increased roughness of the Au film is expected to dramatically affect the magnitude of the electromagnetic enhancement and, therefore, the extent of the SERS effect for these substrates. The scanning electron microscopy (SEM) image of the Au film on glass (Figure S5a) showed a steady corrugated surface with small deposits that were ∼50 nm in diameter. Corrugation and layer growth of Au films on glass have been extensively studied since the early report of the process of obtaining metallic films by evaporation.^42^ The presence of large clusters on the layers formed on the glass substrate was related to the lower deposition rate in the initial stages of layer growth, which leads to discontinuous Au layers, i.e., sites with different probabilities of gold atom capture.^36^

The enhanced surface roughness of Au films on glass produced a strong SERS effect, revealing the bands in the Kerr-gated Raman spectrum from NR in solution (Figure 2c-i). Conversely, the weaker enhancement from the substrate became evident in the case of the less rough Au wafer (Figure 2c-ii), and only a negligible signal was found when Au foil was used (Figure 2c-iii). This trend is in good agreement with decreased roughness (Figure S4) and, therefore, the reduced SERS activity of the substrate. Nevertheless, compared to conventional Raman spectroscopy (Figure 2a, orange), the fluorescence rejection of Kerr gating techniques combined with the SERS effect was effective for observation of the Raman bands. To further expand the surface generality of Kerr-gated Raman spectroscopy to nonplasmonic substrates (under a 633 nm excitation line), Al and Cu foils were used as the substrate material. Therein, no Raman bands for NR were observed when employing either Al or Cu foil, and both materials were confirmed to be non-SERS-active under the 633 nm excitation line (spectra iv and v, respectively, of Figure 2c).

A layer of SHINs was drop-cast onto the five substrates under study, and the resulting Kerr-gated Raman spectra of the NR in solution are shown in Figure 2d. For the Au film substrate, the spectrum obtained with SHINs (Figure 2d-i) was essentially identical to that measured without SHINs (Figure 2c-i), which indicates the negligible combined contribution of the plasmonic NPs to the enhancement of the Raman signal. The lack of synergy to produce a further enhancement effect could be related to the non-uniform deposition of SHINs by drop-casting and how the SHINs are distributed across the thin Au film, combined with the raster scanning conditions of spectral collection. For the Au wafer, the contribution of the plasmonic NPs to the enhancement of the NR signals became more noticeable (Figure 2d-ii) compared with the weaker intrinsic enhancement of the naked Au wafer substrate. The relative contribution from the plasmonic NPs to the enhancement of the Raman signal became even more evident for the non-SERS-active substrates, i.e., quasi-optically flat Au foil (Figure 2c-iii) and the Cu and Al foils (spectra iv and v, respectively, of Figure 2c).

In Kerr-gated Raman spectroscopy, the theoretical enhancement of the Raman signal contrast relative to the background in the total signal (*f*_R/b_) will be determined by the fluorescence lifetime following eq 1(26)
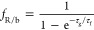
1where *τ*_g_ is the gate time width and *τ*_f_ is the fluorescence lifetime. The gate time width was measured to be approximately 4.5 ps (full width at half-maximum). By considering the fluorescence lifetime of NR in acetone (4.66 ns),^43^ an enhancement factor *f*_R/b_ of 1036 was estimated for the effective suppression of fluorescence, and bands at 586 and 1494 cm^–1^ were likewise identified. Bands in the middle range of the spectra became more apparent (Figure 2d-iii–v), also as a consequence of the electromagnetic coupling in the junction (<10 nm) of two adjacent plasmonic nanoparticles and the substrate (under 633 nm excitation for NPs with an average diameter of 55 nm).^44^

The data in Figure 2 demonstrate effective rejection of fluorescence and Raman signal enhancement for solutions containing 30 mM NR in acetone in a cell with an optical path length of 100 μm. Further experiments were carried out in the absence of a substrate and with a wider optical path. Cuvettes containing 500 μL of a solution (0.2 cm optical path length) were used with solutions of different concentrations of NR (Figure S6). The Kerr-gated Raman spectrum of the solvent was also included for the sake of clarity. For relatively higher concentrated solutions (1 mM NR), the spectrum showed a combination of bands associated with both NR and acetone. For lower concentrations, no bands from NR were detected and only those from the solvent were detected, which further corroborates the requirement of a Raman scattering enhancer in the detection of NR in solution.

These results demonstrate the advantageous association of Kerr-gated Raman spectroscopy with SERS, which includes the use of plasmonic NPs. Nevertheless, the location of the target analyte with respect to the plasmonic hot spots (locations at metal nanostructures with a strong near-field enhancement) is a critical parameter to consider in the amplification of weak Raman signals. Wider gaps between the plasmonic NPs and the substrate were tested by placing the NR solution between two CaF_2_ windows, one containing the NPs and the other the Au foil substrate (Figure S7). The spectrum was similar to the bare substrate spectrum but showed a weak feature at 586 cm^–1^, assigned to ring II breathing (Table S2).

Nanostructured SERS substrates must meet certain requirements for improving the Raman signal and for quenching fluorescence. The shape and size of the nanostructures will govern the formation of hot spots, and the roughness of the substrate will determine the magnitude and range of the enhancement. The distance and orientation of the molecule to the substrate/NPs and matching of laser excitation wavelength play additional crucial roles in both cases.^45^

Au films with SHINs were observed by SEM before and after laser exposure in the Kerr-gated experiment (Figure S5). The bare Au film showed an irregular rough surface with random spacing between heights and depths (Figure S5a). Protuberances on the nanometer scale that resembled dropped Au NPs, which may be the reason for the exceptional signal enhancement, remained unaltered after exposure to a laser (Figure S5c). The surface showed major differences when NPs were deposited on the Au film (Figure S5b), which showed good dispersion on the substrate with no evidence of the formation of large aggregates. Several physical and chemical phenomena can occur during the interaction of lasers with plasmonic NPs, among which is heating of the NP after selective absorption of radiation. A high-temperature boost may lead to particle destruction via coherent acoustic lattice vibrations, melting and evaporation from the particle surface, and explosive fragmentation.^46,47^ After beam exposure, SHINs did not appear to be damaged (Figure S5d). This was further corroborated by transmission electron microscopy images that show that the silica shell remained intact, and no evidence of core melting was found (Figure S1c).

Nile blue [NB, 5-amino-9-(diethylamino)benzo[*a*]phenoxazin-7-ium chloride] in ethanol is expected to show the largest fluorescence background when the laser excitation is close to the maximum of molecular absorption (Figure S3a, blue). Under a 633 nm excitation line (or resonance Raman scattering conditions), NB approaches the most challenging cases of good QY (22%),^48^ and it was used to evaluate and/or correlate the contribution of the substrate and the plasmonic NPs to the enhancement of the Raman signal. The relatively short fluorescence lifetime of NB in ethanol (1.42 ns)^49^ gives a Kerr-gated Raman contrast value close to 316, which was expected to contribute to the difficulty of a direct measurement.

For Au substrates (Figure 3), corrugated surfaces (Au film and Au wafer Figure 3-i,ii) provided enough signal enhancement to allow the detection of NB in solution (10 nM), and the assigned Raman band frequency–vibration correlations are described in Table S2. The effect of SHINs as electromagnetic resonators gained importance for optically flat Au foil substrates (Figure 3-iii). SHINs also provided enhancement of the Raman signal in the case of Al and Cu films (Figure 3-iv,v). In all cases, bands from the solvent were identified (Figure S6 and Table S3), which became more evident for Al and Cu substrates with SHINs together with an increased signal from the background.

**Figure 3:**
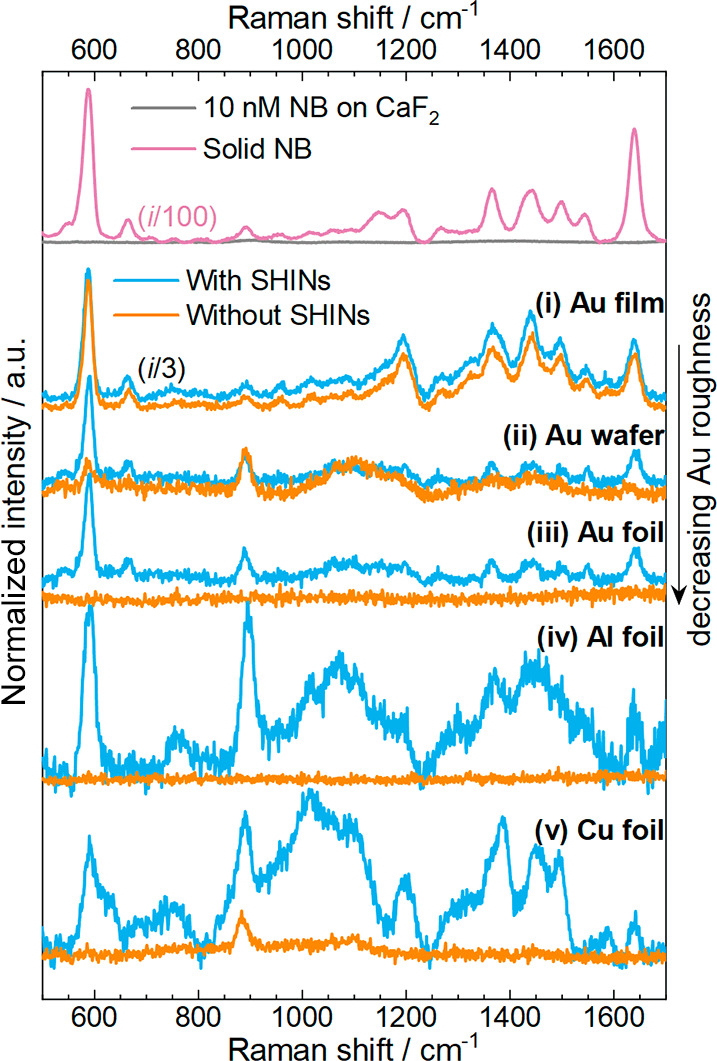
Kerr-gated resonance Raman spectra for solid NB (pink), a 10 nM solution of NB in ethanol on a naked glass window (gray), and 10 nM solutions of NB in ethanol in different substrates without SHINs (orange) and with SHINs (blue): Au film, Au wafer, Au foil, Al foil, and Cu foil. The intensity of spectra from Au film has been reduced by 3-fold, and all spectra were recorded under a 633 nm excitation line with picosecond pulsed laser excitation.

For comparatively more highly concentrated solutions of NR and NB in a 500 μL cuvette (0.2 cm optical path length), the Raman spectra showed bands from both the analyte and the solvent (Figure S6). Raman signals from NR and NB became less obvious at low concentrations until only Raman signals from the solvent were found. These results show that Raman signal enhancers were required for the acquisition of well-resolved Raman spectra, along with efficient rejection of fluorescence at low concentrations.

The spectrum of NB (solid) under conventional CW Raman spectroscopy (Figure S8a, black) was comparable, from the point of view of resolution, to that acquired under the Kerr-gated configuration (Figure 3, pink). However, bands from NB in solution were obscured under the strong background from fluorescence (Figure S8a, blue). The same trend was observed when a non-SERS substrate was used under conventional Raman spectroscopy (Figure S8b). An Al foil substrate with and without a layer of SHINs (Figure S8b, dark blue and light blue, respectively) was used for the collection of the Raman spectra of a solution containing 10 nM NB in ethanol. The signal in both spectra originated essentially from fluorescence. Likewise, the CW Raman spectrum of 10 nM NB in ethanol on a Au film (Figure S8c) led to a large emission baseline, swamping the vast majority of Raman bands and making reliable characterization by CW Raman spectroscopy impossible. These observations demonstrate that fluorescence rejection techniques are required for the detection of fluorophores under resonant conditions, even with SERS substrates. When using Kerr-gated Raman spectroscopy, but in the absence of SERS substrates, mainly the fluorescence background was observed (Figure S8d, light blue) because of the weak Raman signals of NB in solution under a 633 nm excitation line.^48,50^ Consequently, the favorable combination of SERS and Kerr-gated Raman spectroscopy for the system under study is demonstrated in the spectra of a Au film and glass window, in the absence of a NB solution (Figure S8d, black and gray, respectively).

In some instances, for the measurements of NR and NB dyes (Figures 2c,d and 3-iv,v), broad features in the region of 900–1200 cm^–1^ are recorded. It is noted that a majority of these features overlap with minor bands for the NR and NB dyes and solvents used (as summarized in Figure S9). However, the band shapes, relative intensities, and noncorrelative appearances do not support obvious assignments to either dye molecules or solvents. It is observed that these features, while not appearing consistently from sample to sample, are observed only in instances in which the surface enhancement effect is pronounced (evidenced by the observation of target Raman bands in the same spectra). However, their precise assignments are not fully understood.

The successful synergism between fluorescence rejection techniques and Raman signal enhancers has been shown to provide deep insight into systems in which fluorescent species are produced as reaction intermediates and/or (sub)products. This could be the case for fluorescing species formed by decomposition reactions of the electrolyte and electrodes during the cycling of Li-ion batteries. Additionally, it may be highly relevant in the study of reaction mechanisms in the investigation of renewable energy sources (methanol-to-hydrocarbons reaction) and, especially, in the precise detection of target analytes in organelles and cellular structures stained with fluorescent dyes.

In summary, the combination of surface enhancement effects with Kerr-gated Raman spectroscopy for the detection of highly fluorescent samples with weak Raman signals has been demonstrated. This approach mitigates some of the primary drawbacks of Raman spectroscopy, i.e., weakness of the signal and interference from sample-induced fluorescence emission. The fluorescence lifetimes of molecules adsorbed on metallic surfaces may be shortened through concentration quenching or energy transfer quenching compared to conventional low concentrations in solution,^51^ ultimately decreasing the signal-to-noise ratios of this technique. However, comparative measurements excluding either the fluorescence rejection (by the Kerr gate) or the surface enhancement effects resulted in spectra void of useful spectroscopic Raman bands, demonstrating the efficacy of combining the two methodologies.

Therein, fluorescence rejection in the spectra of NR and NB dyes was achieved by using Kerr-gated Raman spectroscopy, but a signal enhancer was necessary to observe the Raman bands. It was shown that SERS effects were induced with the 633 nm picosecond pulsed laser excitation when utilizing a Au substrate of sufficient surface roughness. Coupling this effect with the Kerr gating configuration yielded well-resolved spectra with high signal-to-noise ratios by effective rejection of the competing fluorescence emission signals. Corresponding weaker signal enhancements correlated well with the lower surface roughness of the different Au substrates used, as supported by SEM and electrochemical measurements.

With regard to non-SERS-active substrates, optically flat Au, Al and Cu foils, it was demonstrated that shell-isolated nanoparticles could be exploited as the electromagnetic resonators to enhance the Raman signals from the dyes using Kerr-gated Raman spectroscopy. In all cases, neither the SERS substrates nor the coated nanoparticles showed any sign of degradation after exposure to the lasers in the Kerr-gated Raman configuration.

Correlative Kerr-gated SERS has the potential to screen analytes, products, and reaction intermediates that have been previously obscured by the emission background, in fields as diverse as biosensing, catalysis, and energy materials.

## Supporting Information Available

Experimental details, characterization of SHIN particles before and after laser exposure, ultraviolet–visible (UV–vis) absorption spectra of SHINs on glass and on Au films, UV–vis absorption spectra and chemical structures of the dyes used, electrochemistry of different Au substrates, SEM images of deposited SHINs before and after laser exposure, additional Kerr-gated Raman spectra of dye solutions, and Raman band assignment tables for the various reported spectra (PDF)
