# Supplementary material for: Simultaneous Surface-Enhanced Raman Scattering with a Kerr Gate for Fluorescence Suppression
Source: J Phys Chem Lett. 2024 Jan 10;15(2):608–15. doi: 10.1021/acs.jpclett.3c02926 (PMC10801684; doi:10.1021/acs.jpclett.3c02926)
Supplement: Supplementary file 1 — jz3c02926_si_001.pdf [file jz3c02926_si_001.pdf]

Supporting Information.

Simultaneous Surface Enhanced Raman Scattering with a Kerr-Gate for Fluorescence Suppression

Gema Cabello,[*,‡,a,b] Igor V. Sazanovich,[c] Ioannis Siachos,[d] Matthew Bilton,[e] Beata L. Mehdi,[d] Alex R. Neale,[a,b] Laurence J. Hardwick[*,a,b]

[a] Stephenson Institute for Renewable Energy, Department of Chemistry, University of Liverpool, Peach Street, Liverpool, L69 7ZF, UK

[b] The Faraday Institution, Quad One, Harwell Science and Innovation Campus, Didcot, UK

[c] Central Laser Facility, Research Complex at Harwell, STFC Rutherford Appleton Laboratory, Harwell Campus, Didcot, Oxfordshire, UK

[d] Department of Mechanical, Materials and Aerospace Engineering, University of Liverpool, Liverpool L69 3GH, UK, England, UK

[e] SEM Shared Research Facility, University of Liverpool, Liverpool, L69 3GH, UK

Corresponding authors:

gcabello@slb.com; hardwick@liverpool.ac.uk

Experimental

Instrumentation

Continuous wave Raman spectra were recorded with an inVia Raman (Renishaw) spectrometer equipped with thermoelectrically cooled (−70 °C) CCD camera and a confocal Leica microscope. A He-Ne laser beam at 633 nm, 50×/0.75 NA objective lens and 1800 lines/mm gratings were used. Laser power at the sample surface was restricted below 0.01 mW. All spectra were recorded using 10 s integration time, 1 scan accumulation and were corrected by polynomial function background subtraction. No smoothing function was applied.

Kerr-gated Raman measurements were carried out at ULTRA laser facilities at Central Laser Facility (Science and Technology Facilities Council [STFC], Rutherford Appleton Laboratories, UK) and the experimental set-up has been described previously.[1] Herein, Kerr-gated Raman scattering was achieved under 633 nm laser pulse (2.6 mW, 10 kHz, 2 ps) with a 75 x 75 μm^2 spot size. The Kerr medium was activated by a gating pulse (800 nm, 240 μJ, 2 ps, 10 kHz). All spectra were collected and averaged over 4 repeats, each with an acquisition time of 60 s. The slit size was 300 µm and the spectra were collected under rastering conditions. The Raman shift was calibrated against the spectrum obtained for toluene.

UV/vis extinction/absorption spectra were collected in the range from 350 to 700 nm with an Evolution 201 UV/Visible spectrometer (Thermo Fisher Scientific). Transmission electron microscopy (TEM) was used for the characterization of the morphology of the NP and thickness of the coating using a JEM-2100 Plus (JEOL) operated at 200kV accelerating voltage. Scanning electron microscopy (SEM) images were acquired with a JSM 7001F FEG-SEM (JEOL).

A conventional three-electrode electrochemical cell was used for the electrochemical studies. Charge measurements were carried out from the first scan CV using a VSP3 potentiostat (Biologic Science Instruments). The counter electrode was a gold wire of

sufficiently large area, and all the potentials were measured against the Ag/AgCl (saturated KCl) reference electrode. The electrolyte was 0.5 M H_2SO_4 prepared in Milli-Q water (Millipore). All the experiments were conducted in an atmosphere of oxygen-free N_2 gas using a sweep rate of 50 mV s^{-1} and scan range from -0.2 V to 1.3 V.

Materials & methods

All reagents were acquired from Sigma Aldrich in the maximum purity available (ACS reagent grade) and used without further purification.

Substrate identification and preparation

Five materials were used as substrates for the spectroscopic measurements in this work, in addition to microscope cover and their respective labels, and methods for preparation, are described below.

"**Au film**" - Au films (100 nm thick) were deposited onto microscope cover glass by thermal evaporation of Au wire (99.999%, Advent) with an Oerlikon Leybold Vacuum Univex 300.

"Au on wafer" - Au-coated (50nm layer thickness) Si wafers (Platypus Tech) were cut into 0.5 x 0.5 cm^2 pieces, cleaned with piranha solution, rinsed with Milli-Q water, and dried with N_2 before use.

"**Au foil**" - Au foil (0.02 mm thickness on 5 µm polyester support, 99.9%, Goodfellow Cambridge Ltd.), was manually polished with alumina suspension down to 0.05 µm-particle and further sonicated in acetone and Milli-Q water before use.

"Al foil" and **"Cu foil" -** Al and Cu foils (0.035 mm thickness) were acquired from RS Components and used without processing.

SHIN synthesis and preparation procedure

Silica-coated Au NPs (SHell-Isolated Nanoparticles, SHINs) were synthesized following the method described elsewhere.[2] Briefly, 55 nm Au NPs were synthesized by chemical reduction of $HAuCl_4.3H_2O$ with sodium citrate.[3, 4] The citrate stabilizing layer was then replaced by (3-aminopropyl)triethoxysilane and subsequently by a silica shell, under strict control of pH and temperature.[5] The effective coating of the Au core was demonstrated by the conventional pinhole test using 10 mM pyridine (99.8%, Sigma-Aldrich) as indicator.[2] For the spectroscopic measurements involving SHINs, a layer of the SHINs was deposited onto the substrate under investigation by drop-casting 20 µL of concentrated SHINs dispersion. The substrate was then dried under gentle vacuum conditions to remove water.

Chemisorbed Rhodamine 6G (Rh6G) preparation

For the spectra presented in Figure 1 [a(i), a(ii) and b], the Au film substrate was immersed in a solution of Rh6G in ethanol for 5 h, then rinsed with H_2O (high purity) and dried under N_2 before measurements. For spectra in Figure 1 (a), the concentration of Rh6G in ethanol was 100 µM, whereas for Figure 1 (b) the concentration of the Rh6G solution is labelled (100 µM, 10 µM, 1 µM).

Supporting figures and data

Characterization of NPs

In SHINER spectroscopy, the structural parameters of the plasmonic core will determine the efficiency of the surface enhancement and, therefore, the enhancement of the signal in the vibrational spectrum of target analytes. Specifically, the wavelength corresponding to the localized surface plasmon resonance (LSPR) is conditioned by the nature of the metal, shape, size and degree of aggregation of the nanoparticles.[6] The plasmonic resonance extinction peaks of 50 nm (average diameter) Au NPs (black) and SiO_2-coated Au NPs (SHINs, grey) are shown in Figure S1a. Au showed resonant behavior when interacting with ultraviolet and visible (UV–vis) photons and the silica shell did not affect significantly the optical properties of Au NPs. The SiO_2 shell, required to chemically isolate the metallic core and inhibit surface chemical or electrical interactions with the analyte needs to be robust as well as sufficiently thin as to preserve the relative short-range effect of the plasmonic electromagnetic field. Herein, the shell thickness for the as-prepared SHINs was found to be uniform with approximately 3 nm thickness (Figure S1b).

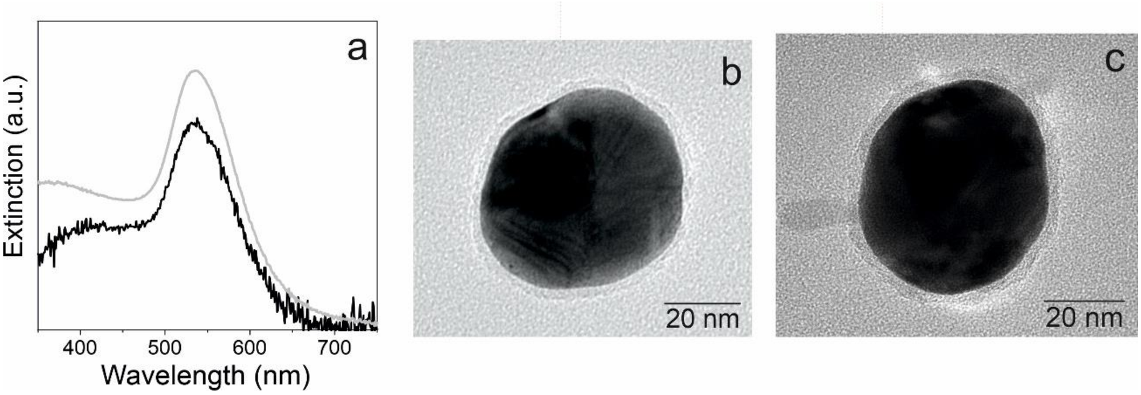

Figure S1. Extinction spectra of 50 nm Au NPs (black) and SHINs (grey) showing their respective plasmonic resonance extinction peaks (a). High-resolution TEM image of a single 55nm Au nanoparticle coated with a 3 nm SiO_2 layer, before and after exposure to Kerr-gated Raman laser (b & c, respectively).

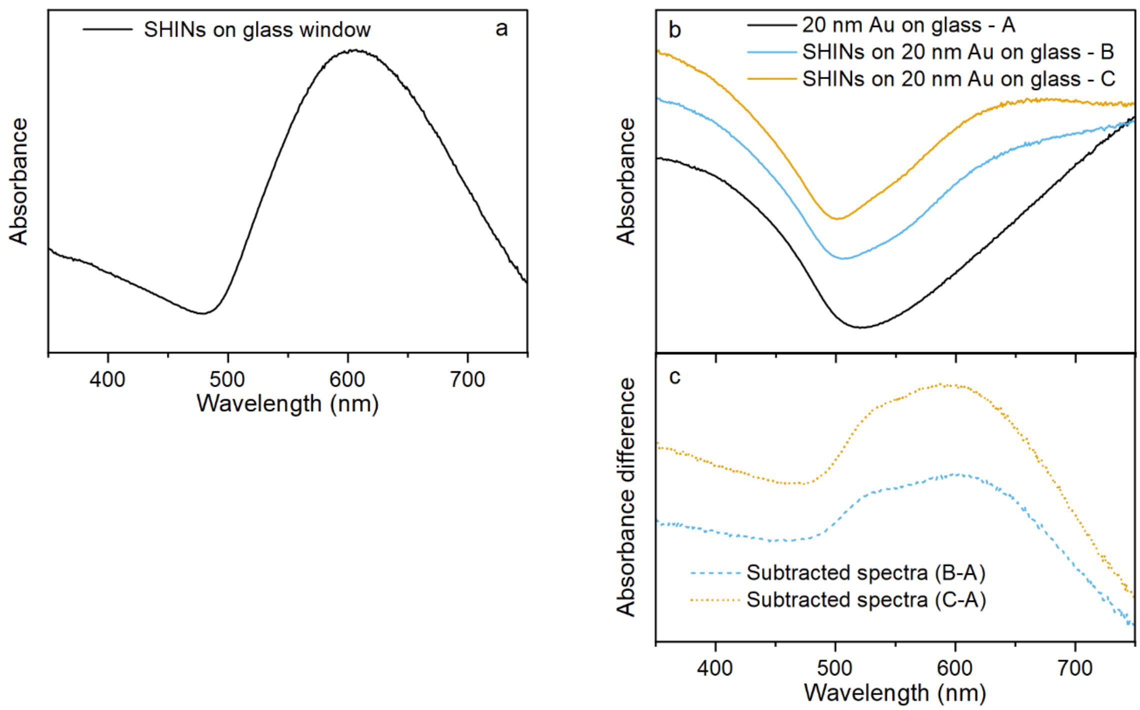

Figure S2. UV-vis absorbance spectra of (a) SHINs deposited on a glass microscope slide and of (b) a glass microscope slide with a Au film (20 nm) without (black trace) and with (blue and orange traces) layers of SHINs. The difference spectra (c) for the Au layer with and without the addition of SHINs was calculated by subtraction of the Au film only spectrum (A) from the spectra of SHINs plus Au film (B and C).

To achieve measurable absorption contributions from SHINs, these were deposited using multiple drops from a concentrated dispersion of SHINs. Additionally, to enable transmission mode measurement of UV-visible absorption spectra of Au films analogous to those used in the Kerr-gated Raman measurements, thinner (20 nm) Au layers were deposited to permit a reasonable optical transparency. The spectra B and C in panel (b) were collected at slightly different regions of the microscope slides (accounting for the non-uniform distribution of SHINs across the Au surface).

UV-Vis and chemical structures of dyes

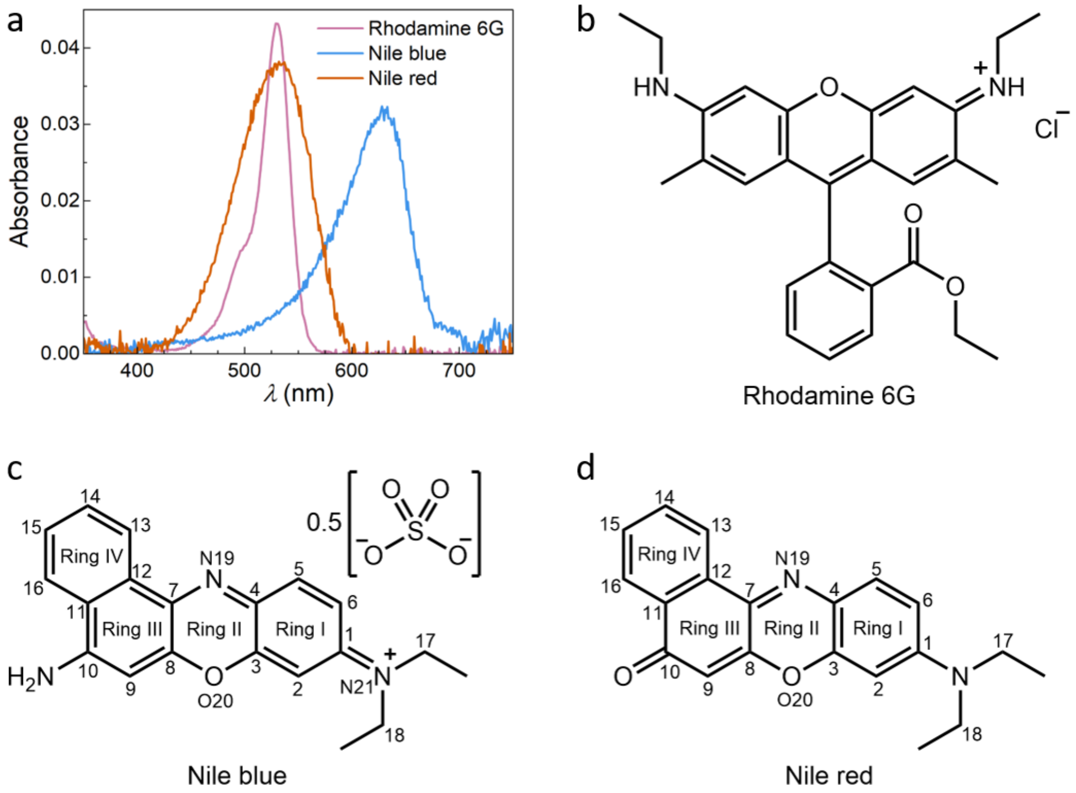

Figure S3. (a) Absorption spectra of rhodamine 6G (pink), Nile blue (blue) in ethanol and Nile red (red) in acetone. (b-d) Corresponding molecular structures of (b) rhodamine 6G, (c) Nile blue, and (d) Nile red.

Electrochemical roughness characterization

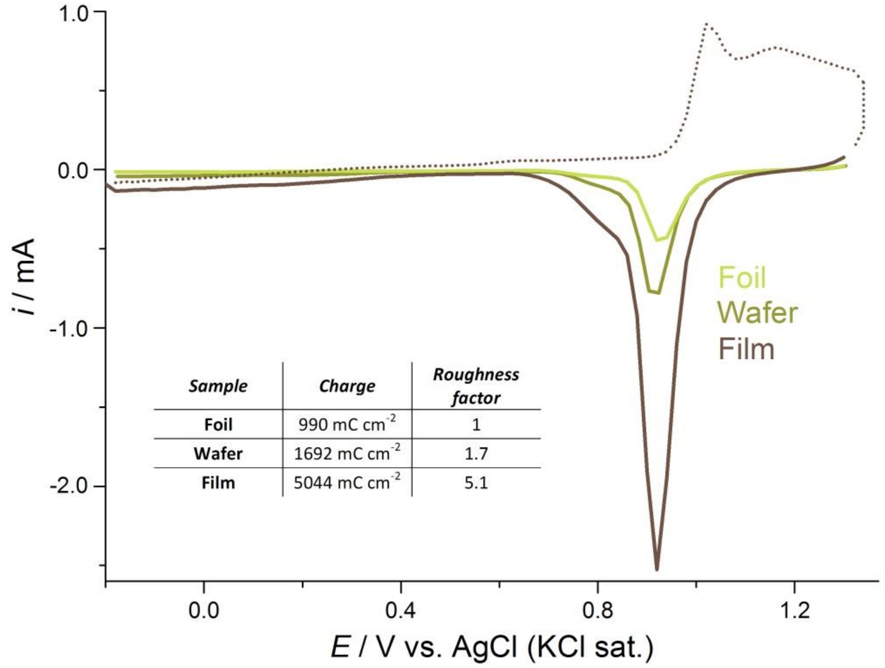

Sample	*Charge*	*Roughness factor*
**Foil**	990 mC cm^{-2}	1
**Wafer**	1692 mC cm^{-2}	1.7
**Film**	5044 mC cm^{-2}	5.1

Figure S4. Cyclic voltammograms of different Au substrates in 0.5 M H_2SO_4 (ΔE = -0.2 – 1.35 V) at 50 mV s^{-1}. The geometrical area was 1 x 1 cm^2 in all cases. The areal charge associated with the cathodic peak at *ca.* -0.9 V, and the derived roughness factor, are provided in the table.

Characterization of Au film substrate and SHINs

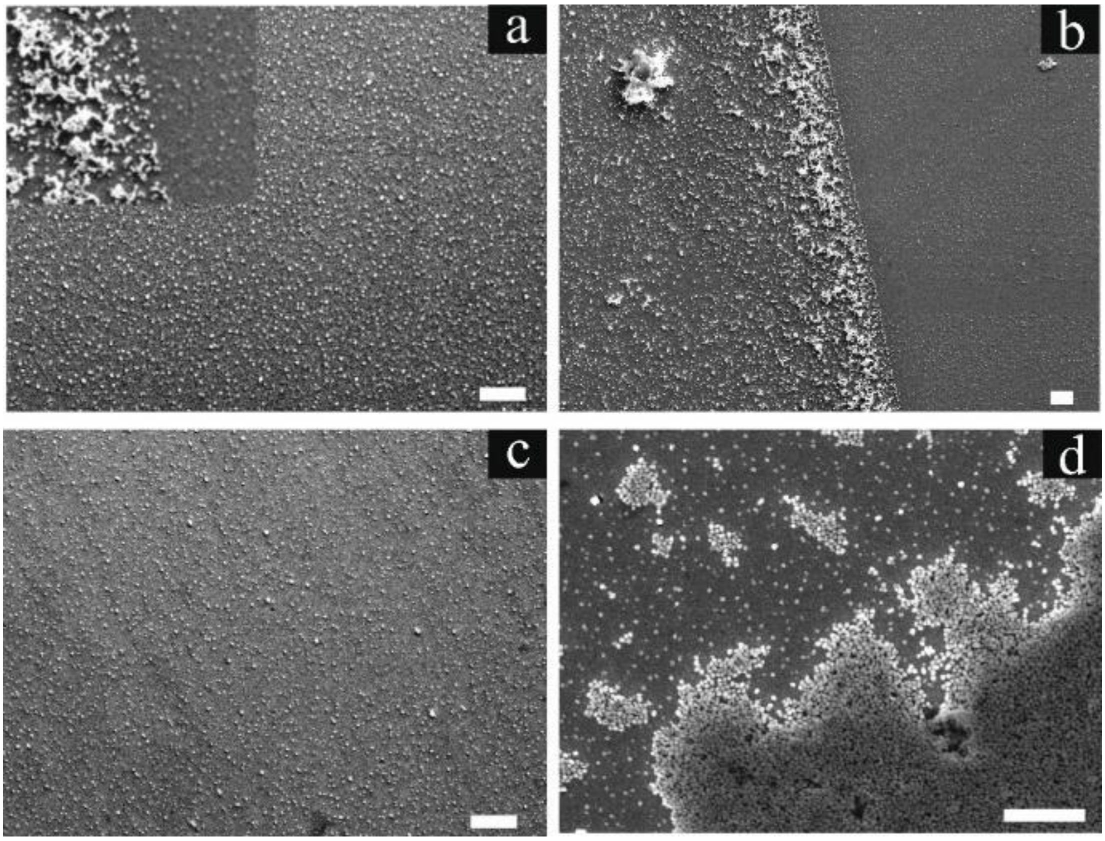

Figure S5. Representative SEM images of bare Au film (left) and SHINs-coated Au film (right) before (a & b) and after (c & d) exposure to the Kerr-gate laser (633 nm, 2.6 mW, 12 min). Scale bar = 1 μm.

Kerr-gated Raman spectra of bulk dye solutions

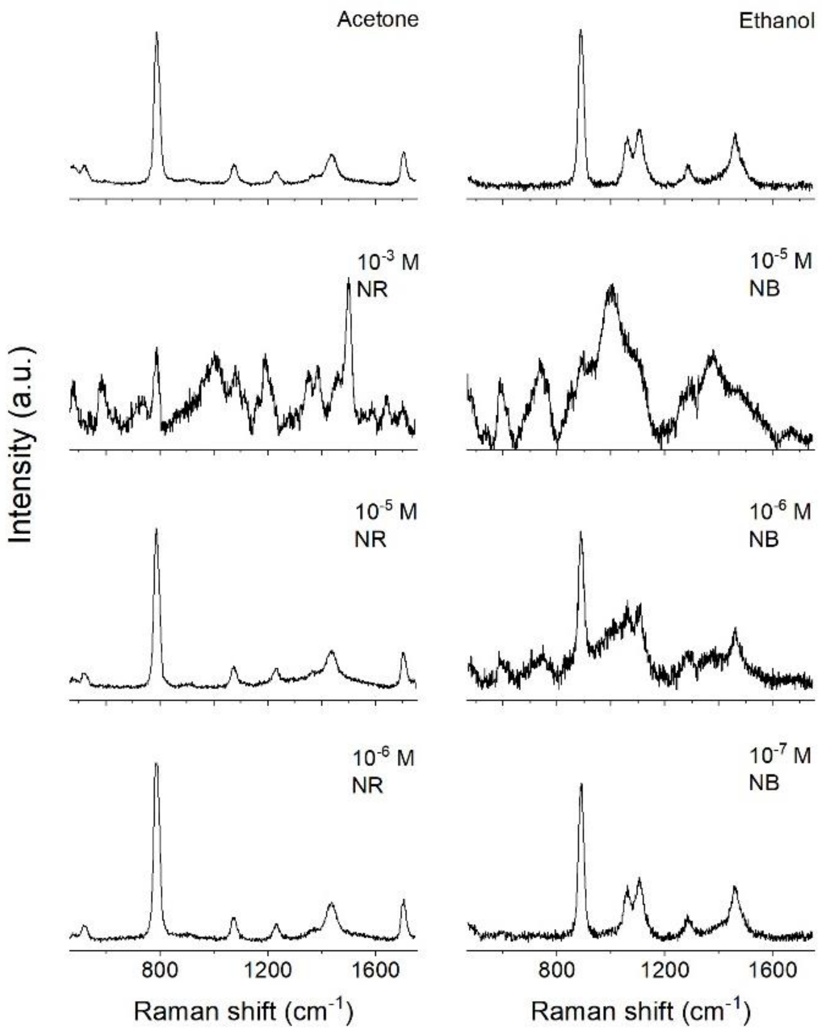

Figure S6. Kerr-gated Raman spectra of acetone and solutions of Nile red (NR) in acetone at different concentration (panels left) and ethanol and solutions of Nile blue (NB) in ethanol at different concentration (panels right). All performed using a cuvette, containing 500 μL solution (0.2 cm optical path).

Cell configurations for SHIN-enhanced Kerr-gated Raman measurements

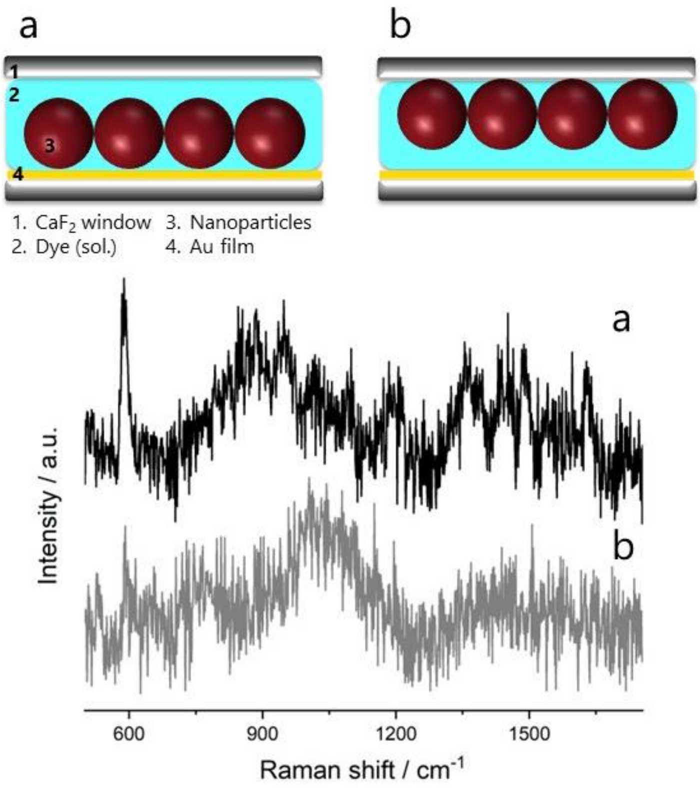

Figure S7. Different cell configurations used to acquire the Kerr-gated Raman spectra of a solution containing 30 nM Nile Red in acetone on Au foil substrate: SHINs on Au foil (a) and SHINs on cover-CaF_2 window (b).

Continuous wave and Kerr-gated Raman spectra of Nile blue

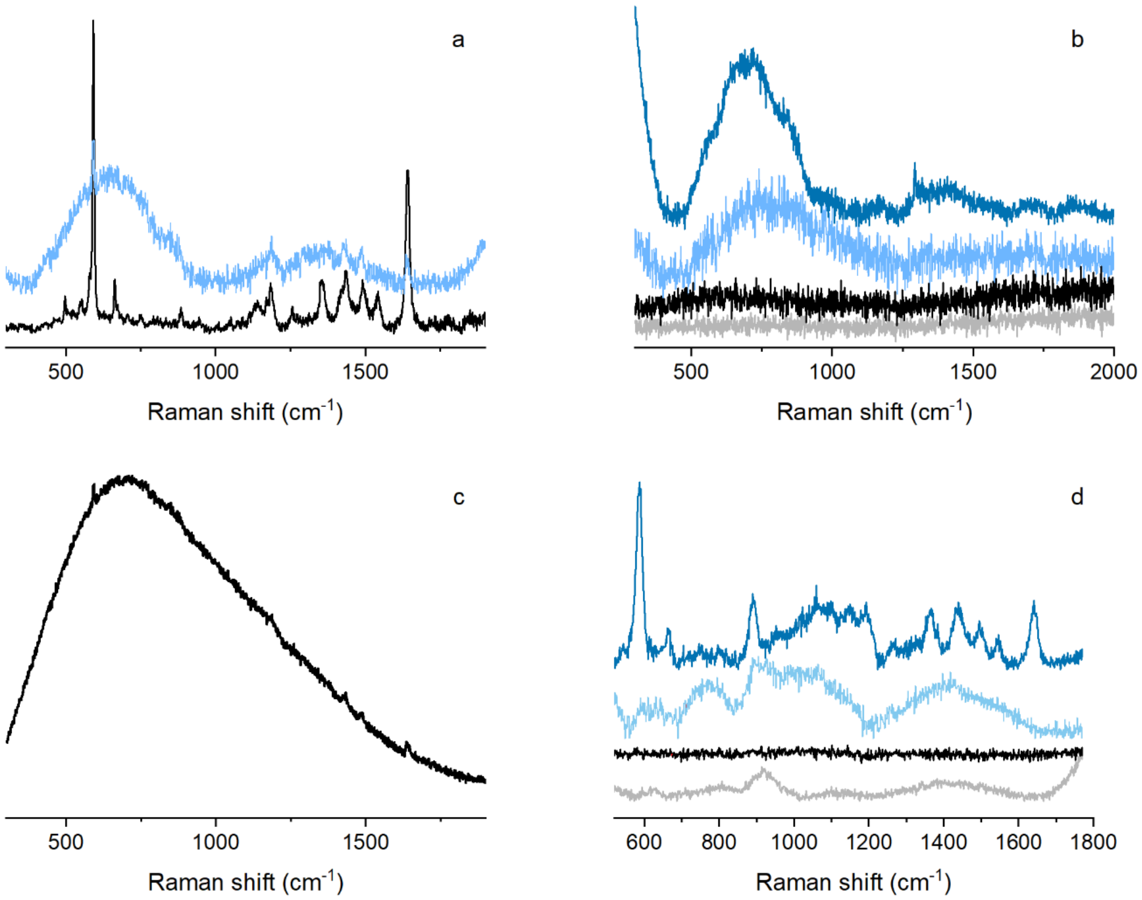

Figure S8. (a) Continuous wave (CW) Raman spectra of solid Nile blue (NB) (black trace) and a solution containing 10 nM NB in ethanol (blue trace). (b) CW Raman spectra of Al foil: bare (grey trace); with a layer of SHINs (black trace); with a solution containing 10 nM NB in ethanol (light blue trace); with a layer of SHINs and a solution containing 10^{-5} M NB in ethanol (dark blue trace). (c) CW Raman spectra of 10^{-5} M NB solution in ethanol on a Au film substrate. (d) Kerr-gated Raman spectra of 10 nM NB in ethanol on a gold film evaporated on a glass window and covered with a CaF_2 window (dark blue trace); between 2 glass windows (with NB, no Au film, (light blue trace); between two CaF_2 windows (no NB, with Au film, black trace) and Kerr-gated Raman spectrum of a glass window (no NB, no Au film (grey trace)). All spectra were recorded under 633 nm excitation line.

Unassigned broad features in enhanced Kerr-gated Raman spectra

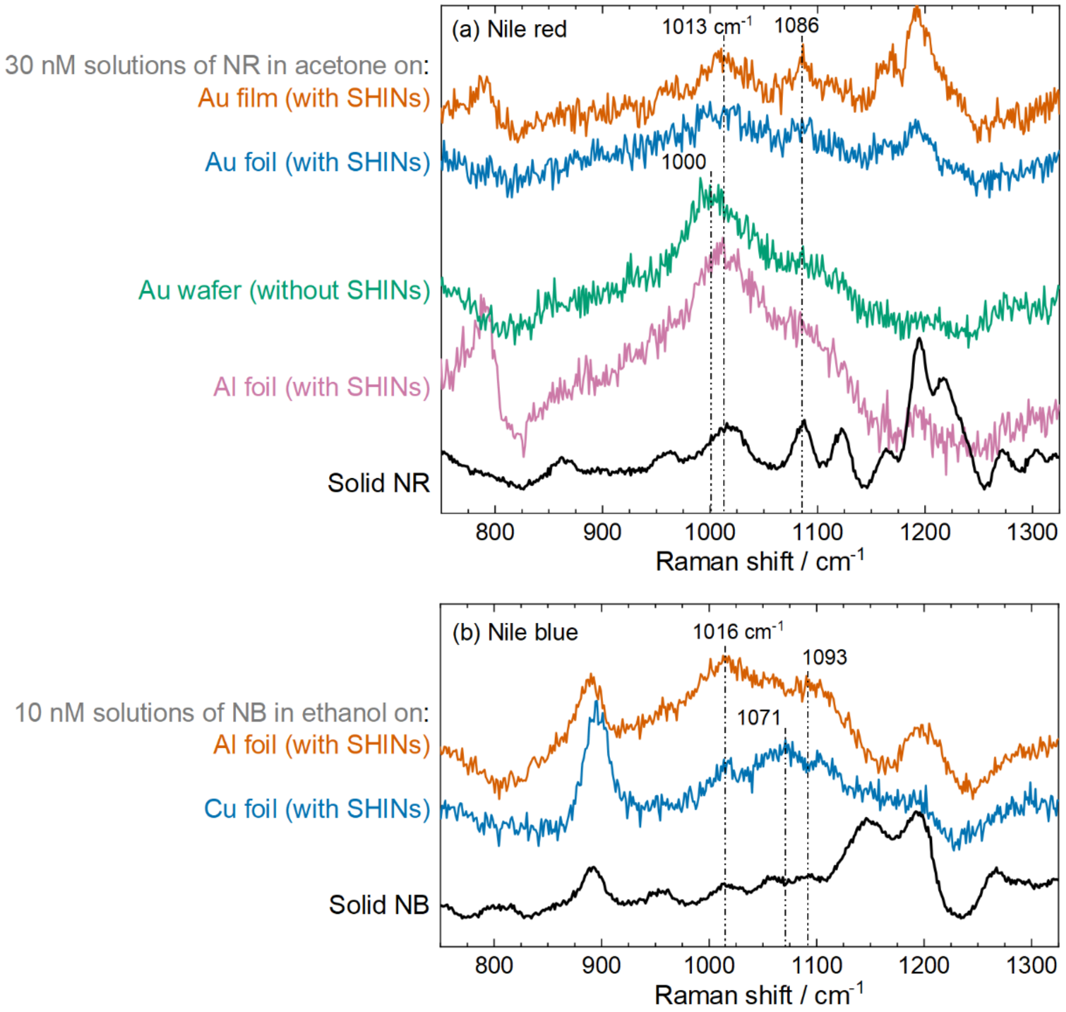

Figure S9. Comparison of additional broad features found in Kerr-gated Raman spectra with enhancement effects for Nile red (a) and Nile blue (b) solutions at different substrates compared with the Kerr-gated Raman spectra of the solid samples (black traces).

The data presented here is a redrawing of select data presented originally in Figure 2 and Figure 3 where broad features in the 900-1200 cm^{-1} regions were observed. The dashed lines, and associated labels (in wavenumbers), highlight the apparent primary band maximum positions and their overlap with minor bands associated with the pure dye molecules (black traces in each panel). It is noted that the band shapes, relative intensities, and inconsistent appearance make reliable assignments to the dye molecules (or solvents) a challenge and their precise assignments are not fully understood.

Raman band assignments

Table S1. Raman band frequencies (in cm^{-1}) obtained from Kerr gated Raman spectrum of 30 nM Nile red (NR) in acetone on evaporated Au film.

505 cm^{-1} (w)	β(C-C-C) rings III & IV + β(C-N21-C)
543 cm^{-1} (vw)	β(C-N21-C-C) + β(C-C-C) ring IV
586 cm^{-1} (m)	Ring II breathing + β(C-CO-C) ring III
643 cm^{-1} (w)	β(C-C) ring IV + ν_{as}(C-C) ring I
719 cm^{-1} (m)	ν(C-C) + ν(C-O-C) ring II + δ(C-H)
747 cm^{-1} (m)	δ(C-H)
861 cm^{-1} (w)	ν(C-CO-C) ring III + δ(C-H)
1017 cm^{-1} (m)	β(C-H)
1087 cm^{-1} (m)	ν(C-O-C) ring II + β(C-H)
1123 cm^{-1} (m)	β(C-H)
1193 cm^{-1} (s)	β(C-H) ring IV + ν(C-N-C) ring II + β(C-H) ring I
1217 cm^{-1} (s)	β(C-H) ring IV + β(C11-16) + β(C9-H) + ν(C-N) ring II
1274 cm^{-1} (w)	β(C-H) ring IV + ν(C10-11) ring III + ν(C-N) ring II + ν(C-N21-C) ring II
1350 cm^{-1} (s)	ν(C-C) ring IV + ν_{as}(C-N19-C) ring II + β(C-H) rings I & III
1387 cm^{-1} (s)	ν(C-C) rings I and III + ν(C-N19-C) ring II
1451 cm^{-1} (s)	ν(C-C) ring IV + ν(C-N19-C) ring II + ν(C-N) ring I + ν(C-C) ring I
1494 cm^{-1} (vs)	δ(C17-H) + + ν(C4-N19) ring II + ν(C-C) ring I
1588 cm^{-1} (m)	ν(C-C) ring IV
1642 cm^{-1} (m)	β(C=C) ring I

Abbreviations used: (vs) = very strong; (s) = strong; (m) = middle; (w) = weak. ν = stretching; ν_s = symmetric stretching; ν_{as} = asymmetric stretching; β = in-plane bending.

Table S2. Raman band frequencies (in cm^{-1}) obtained from Kerr-gated Raman spectra of 10 nM Nile blue in ethanol on evaporated Au film.

592 cm^{-1} (vs)	ν_s(C3−O20−C8) + ν_s(C4−N19−C7) [ring-II breathing]
664 cm^{-1} (w)	ip ν_s(C12−C13−C14) + ip ν_s(C11−C16−C15) ring-IV + ν_{as}(C1−C6−C5) ring-I
1151 cm^{-1} (sh)	β(C6-H) + β(C5-H) ring I + β(CH) ring IV + ν_{as}(C4−N19−C7)
1189 cm^{-1} (s)	β(C-H) ring IV + ν_{as}(C4−N19−C7) + β(C-H) ring-I
1294 cm^{-1} (w)	β(C10-H) ring III + β(C-H) ring IV + ν(C10−C11) + ν(C12−C13) + + ν(C4−N19) + ν(C1−C2) + ν_{as}(C18-N21-C17)
1320 cm^{-1} (vw)	ν_s(C4−N19−C7) + β(C-H) ring IV + β(CH) ring III + β(C2-H)
1362 cm^{-1} (s)	ν(C11−C12) + ν(C−C) ring VI + ν(C1−N21) + ν_{as}(C4−N19−C7) + β(C5-H) + β(C9-H)
1425 cm^{-1} (vw)	ν(C−C) ring IV + ν_s(C4−N19−C7) + ν(N21−C1) + ν(C5−C6)
1496 cm^{-1} (s)	δ(C17−H) + ν(C4−N19) + ν(C14−N21) + ν(C4−C3)
1550 cm^{-1} (m)	ν_{as}(N21−C1) + ν(C2−C3) + ν(C4−N19) + ν(C−C) ring I
1585 cm^{-1} (w)	ν(C11−C12) + ν(C14−C15) + ν(C9−C10)
1639 cm^{-1} (s)	ν(C6−C5) + ν(C2−C3) + ν(C1−N21) in-phase [ring I (C=C) stretching]

Abbreviations used: (vs) = very strong; (s) = strong; (m) = middle; (w) = weak. ν = stretching; ν_s = symmetric stretching; ν_{as} = asymmetric stretching; β = in-plane bending; ip = in-plane

Table S3. Raman band frequencies (in cm^{-1}) obtained from Kerr-gated Raman spectra of ethanol.

887 cm^{-1} (vs)	ν(C−C−O)
1060 cm^{-1} (s)	ν(C−O)
1110 cm^{-1} (s)	CH_3 rocking
1292 cm^{-1} (w)	CH_3 scissoring and twisting
1456 cm^{-1} (s)	δ(CH_3)

References

(1) Cabo-Fernandez, L.; Neale, A. R.; Braga, F.; Sazanovich, I. V.; Kostecki, R.; Hardwick, L. J. Kerr gated Raman spectroscopy of LiPF6 salt and LiPF6-based organic carbonate electrolyte for Li-ion batteries. *Phys. Chem. Chem. Phys.* **2019**, *21* (43), 23833-23842.

(2) Li, J. F.; Huang, Y. F.; Ding, Y.; Yang, Z. L.; Li, S. B.; Zhou, X. S.; Fan, F. R.; Zhang, W.; Zhou, Z. Y.; Wu, D. Y.; et al. Shell-isolated nanoparticle-enhanced Raman spectroscopy. *Nature* **2010**, *464* (7287), 392-395.

(3) Turkevich, J.; Stevenson, P. C.; Hillier, J. A study of the nucleation and growth processes in the synthesis of colloidal gold. *Discussions of the Faraday Society* **1951**, *11* (0), 55-75.

(4) Frens, G. Controlled Nucleation for the Regulation of the Particle Size in Monodisperse Gold Suspensions. *Nat. Phys. Sci.* **1973**, *241* (105), 20-22.

(5) Liz-Marzán, L. M.; Giersig, M.; Mulvaney, P. Synthesis of Nanosized Gold−Silica Core−Shell Particles. *Langmuir* **1996**, *12* (18), 4329-4335.

(6) Langer, J.; Jimenez de Aberasturi, D.; Aizpurua, J.; Alvarez-Puebla, R. A.; Auguié, B.; Baumberg, J. J.; Bazan, G. C.; Bell, S. E. J.; Boisen, A.; Brolo, A. G.; et al. Present and Future of Surface-Enhanced Raman Scattering. *ACS Nano* **2020**, *14* (1), 28-117.
